# Le traitement chirurgical des adénocarcinomes de la jonction oesogastrique: Expérience marocaine à travers une série de 149 cas

**DOI:** 10.4314/pamj.v8i1.71150

**Published:** 2011-03-30

**Authors:** Meryem Glaoui, Sarah Naciri, Samia Ghanem, Abdelkader Belkouchi, Hassan Errihani

**Affiliations:** 1 Service d’oncologie medicale, Institut National d’Oncologie, Rabat, Maroc; 2 Service de chirurgie viscérale A, CHU Ibn Sina, Rabat, Maroc

**Keywords:** Cardia, adénocarcinome, Siewert, chirurgie, Maroc

## Abstract

**Introduction:**

L'incidence du cancer de la Jonction oesogastrique (JOG) ne cesse d'augmenter depuis les deux dernières décades aussi bien dans les pays industrialisés que dans les pays en voie de développement notamment le Maroc. Le rapprochement sur le plan étiopathogénique des adénocarcinomes (ADK) du cardia et ceux du bas oesophage reste un sujet de controverse posant le problème du choix thérapeutique chirurgical, notamment l’étendu de la résection. Le but de ce travail est de dresser le profil épidémiologique des patients opérés pour un ADK du cardia et analyser les gestes chirurgicaux réalisés par l’équipe chirurgicale A du centre hospitalier universitaire IBN SINA à Rabat à travers une série de 149 cas.

**Méthodes:**

Il s'agit d'une étude rétrospective ayant intéressé les malades opérés pour un ADK de la JOG sur une période de 15 années (1990-2004) en chirurgie A du CHU IBN SINA à Rabat.

**Résultats:**

149 cas d'ADK de la JOG ont été retenus. L'âge moyen était de 55 ans, 76% étaient de sexe masculin avec un sex-ratio de 3/1. Les signes cliniques les plus fréquemment observés sont la dysphagie (70%), les douleurs épigastriques (67%) et le reflux gastro-oesophagien (15.5%). La notion de tabagisme n’a été rapportée que chez 20% des cas et l'oesophage de barret chez 10% des patients. Le type I de Siewert a concerné 65 cas (43.5%), le type II 40 cas (27 %), et le type III 44 cas (29.5%). Dans le type I une oesophagectomie transhiatale a été proposée, alors que les type II et III ont été traité comme un cancer de l'estomac par une gastrectomie totale. Les suites opératoires étaient simples chez 80 % des patients, la mortalité globale était de 8.5%.

**Conclusion:**

L’oesophagectomie par voie transhiatale chez les patients fragiles avec un ADK de la JOG de type I permet des résultats carcinologiques satisfaisants avec réduction de la morbidité postopératoire par rapport à la voie transthoracique. La gastrectomie totale est le traitement de choix pour les types III, alors que le débat est toujours ouvert quant à la meilleure stratégie chirurgicale pour la prise en charge des tumeurs de type II.

## Introduction

La fréquence des adénocarcinomes de la jonction oesogastrique augmente de façon alarmante, plus rapidement que n’importe quel autre cancer en Europe [1]. Ces derniers différent des cancers épidérmoides de l’oesophage non seulement par l’histologie mais aussi en raison d’une histoire naturelle et pronostic différents [2, 3], Ils différent également des adénocarcinomes gastriques sous jacents par une épidémiologie propre, un envahissement lymphatique précoce, une propagation bidirectionnelle vers le Médiastin et l’abdomen [4] et un pronostic plus sombre [5].

Cette entité a été démembrée par la classification de Siewert et al [6] en 3 types : type I (ou adénocarcinome du bas oesophage, dont le centre est situé 1 cm au dessus de l’épicentre de la tumeur), type II (ou adénocarcinome du cardia anatomique, dont le centre est situé entre 1 cm au dessus et 2 cm en dessous de l’épicentre de la tumeur), et type III (adénocarcinome du fundus étendu au cardia, dont le centre est situé 2 cm en dessous de l’épicentre de la tumeur). Cette classification est utile en termes de choix thérapeutique chirurgical puisque le type I est traité comme un cancer de l’oesophage alors que le type III rejoint le traitement du cancer de l’estomac, le traitement des types II est toujours controversé [7].

Le but de ce travail rétrospectif est de rapporter les caractéristiques épidémiologiques des malades opérés pour un ADK de la JOG et d’analyser les différents gestes chirurgicaux effectués en fonction de la classification de Siewert.

## Méthodes

Il s’agit d’une étude rétrospective ayant intéressé les malades opérés pour un adénocarcinome (ADK) de la jonction oesogastrique (JOG) sur une période de 15 années (1990-2004) en chirurgie A du CHU IBN SINA à Rabat. Nous avons inclus les ADK intéressant le cardia anatomique et dont le centre est situé à moins de 5 cm de celui-ci. L’absence d’un endobrachyoesophage (EBO) ou d’une métaplasie intestinale est facultative puisque l’on considère actuellement que la progression tumorale peut détruire ces lésions. Ont été exclu de cette étude, les carcinomes épidérmoides (CE) et les autres types histologiques, ainsi que les ADK du 1/3 supérieur de l’estomac dont le centre est situé à plus de 5 cm de la JOG. Au total, nous avons retenu 149 cas.

## Résultats

L’étude a porté sur 113 hommes (76%) et 36 femmes (24%), le sex-ratio était de 3/1. L’âge variait de 22 à 84 ans avec un âge médian de 55 ans. Parmi les antécédents des patients inclus dans notre série on note la notion de RGO chez 23 malades (15.5 %), d’EBO chez 16 malades (10.7 %) et d’Intoxication alcoolo tabagique chez 39 malades (26%). Le délai moyen au diagnostic était de 8 mois (1 à 24 mois). Les circonstances de découverte étaient dominés par : la dysphagie (70%), les épigastralgies (67%), l’altération de l’état général (48 %), les hématémèses (31%) et les vomissements (28%).

Le diagnostic a été établi chez tous nos patients sur des biopsies par FOGD. Selon la classification de Siewert ; 65 malades étaient de type I (43.5%), 40 malades de type II (27%) et 44 malades (29.5%) de type III. La tumeur était localisée chez 104 patients, localement avancée chez 29 patients et métastatique chez 17 patients.

Tous les patients considérés comme ayant un stade localisé étaient opérés d’emblée, le taux de résecabilité était de 70%. Dans le type I une oesogastréctomie transhiatale a été proposée, alors que les type II et III ont été traité comme un cancer de l’estomac par une gastrectomie totale .Les différentes interventions réalisées, les voies d’abord ainsi que le type de rétablissement de continuité sont représentées respectivement dans les tableaux 1, 2 et 3. En outre, un geste associé à une exérèse majeure a été réalisé chez 3 malades chez qui une extension locorégionale fut découverte en per-opératoire;  ainsi, une splénectomie isolée a été faite chez 2 malades et une splénopancréatectomie chez 1 malade.

Le curage ganglionnaire le plus souvent réalisé était de type curage D1,5 comportant les ganglions périgastriques du curage D1 et une partie des ganglions du curage D2 (coronaire stomachique, bord gauche de l’artère hépatique commune, artère splénique), ainsi, 23 % de curage de type D1 ont été réalisé et 67% de curage D1,5. Le taux d’exérèse Ro à l’analyse anatomopathologique des pièces opératoires a été de 62 %.

Les suites opératoires ont été simples chez 84 malades réséqués pour ADK du cardia. Une ou plusieurs complications sont survenues chez 20 malades (Tableau 4). Nous avons déploré 8 décès, la mortalité globale a été de 8.5 %, les causes du décès sont dominées par les complications d’ordre respiratoires (tableau 5).

La durée moyenne d’hospitalisation des patients dans notre institution était de 27 jours chez l’ensemble des opérés. Les variables associées à l’allongement de la durée d’hospitalisation sont essentiellement celles qui sont liées aux complications anastomotiques et/ou respiratoires.

## Discussion

Les adénocarcinomes du bas oesophage, du cardia et de l’estomac sont souvent regroupés ou confondus en une seule entité pour des raisons de simplification ou par pragmatisme du fait de leur similitude histologique [8]. Ainsi, la prise en charge thérapeutique de l’adénocarcinome de l’oesophage est voisine de celle des cancers épidermoïdes, alors que celle de l’adénocarcinome du cardia est plutôt assimilée à celle des cancers gastriques.

Les résultats des récentes séries chirurgicales comportant essentiellement ou exclusivement des adénocarcinomes du cardia ont montré que leur pronostic est plus sombre [9].

L’analyse dans la littérature des différents gestes chirurgicaux réalisés dans les ADK de la JOG prend en considération la classification de Siewert. En effet, pour les types I, Un seul essai randomisé a testé la place d’une voie d’abord transthoracique par comparaison à une voie transhiatale pour la réalisation d’une oesogastréctomie chez 220 patients présentant un adénocarcinome du bas oesophage [10]. Avec un suivi en médian de 4,7 ans, l’analyse des résultats anatomopathologiques a montré que la qualité de l’exérèse était équivalente entre les deux techniques (R0 contre R1 contre R2) [11]. La mortalité post opératoire était similaire dans les deux groupes, alors que les complications respiratoires étaient significativement plus élevées dans le groupe avec une voie d’abord transthoracique (57% vs 27%; p12].

Dans notre série, l’oesogastréctomie par voie transhiatale a été l’intervention de choix pour les tumeurs de type I de Siewert, La préférence pour cette intervention revient au fait que la majorité des patients étaient fragiles, dénutris et dont l’état pleuro-pulmonaire n’était pas toujours satisfaisant et l’éviction de la thoracotomie permettrait de réduire la morbidité post opératoire liée aux complications respiratoires.

Pour les ADK de la JOG de type II, le débat est toujours ouvert quant au meilleur traitement chirurgical. Une étude multicentrique française a démontré que le taux d’exérèse R0 après gastrectomie totale pour les ADK de la JOG de type II localement avancés (T3-T4) était inferieur par rapport à une oesogastrectomie polaire supérieure (64,6% vs 78,7%) sans différence significative en terme de morbidité ou de mortalité postopératoire [13, 14].

Quant à l’équipe de Siewert, ils ont proposé pour le type II, une gastrectomie totale avec oesogastréctomie partielle par voie transhiatale. Une oesogastréctomie polaire supérieure par voie transhiatale n’a été proposée que si une exérèse de type R0 ne pouvait être réalisée par voie abdominale seule [6, 15].

Le traitement chirurgical des ADK du cardia de type III est communément apparenté aux tumeurs gastriques et traitées par une gastrectomie totale étendue associée à une résection transhiatale de l’oesophage distal avec une anastomose oesojéjunaleou oesocolique avec coloplastie [16]. Les tumeurs de type III sont de pronostic défavorable par comparaison aux tumeurs de type I et II, de survie après chirurgie équivalente [17].

En termes de curage ganglionnaire pour les adénocarcinomes de la JOG, trois études randomisées n’ont pas permis de mettre en évidence de supériorité en termes de survie du curage ganglionnaire D2 par comparaison au curage D1 (correspondant aux ganglions paracardiaux, de la petite et de la grande courbure, ganglions supra-et infra pyloriques) [18-20]. Néanmoins, les résultats d’une importante étude prospective allemande ayant inclus 1654 patients opérés pour un cancer gastrique avec un curage D1 (moins de 25 ganglions) ou un curage D2 (plus de 25 ganglions) ont démontré que le curage ganglionnaire D2 améliorait significativement la probabilité de survie globale à dix ans de 26 % pour les patients atteints d’une tumeur de stade II [21].

Au terme de ces résultats : l’association française de chirurgie recommande pour le type I de Siewert une oesogastrectomie polaire supérieure par voie transthoracique avec un curage deux champs : médiastinal et abdominal, la voie transhiatale n’étant réservée qu’aux patients fragiles et en cas de contre-indication à la thoracotomie.

Deux approches chirurgicales peuvent être proposées pour les ADK de la JOG de type II: une oesogastréctomie totale par voie abdominale ou une oesogastréctomie polaire supérieure par voie transthoracique ou transhiatale associées à un curage ganglionnaire médiastinal et abdominal.

La gastrectomie totale étant le traitement de choix pour les tumeurs de type III associé à un curage de type D2 sans splénopancreatectomie étant responsable de la morbidité et de la mortalité du curage D2, celle-ci peut être réservée uniquement à la présence de ganglions macroscopiquement envahis le long de l’artère splénique ou dans le cas d’une tumeur envahissant la grosse tubérosité et la séreuse.

## Conclusion

Le choix de la technique chirurgicale dans les ADK de la JOG est controversé. Les résultats de notre série confirment que l’oesogastréctomie par voie transhiatale peut être une alternative intéressante à la thoracotomie chez les malades fragiles avec des résultats carcinologiques similaires. La discussion est toujours ouverte quant à l’étendue de la résection oesophagienne et gastrique pour les tumeurs de type II. L’oesogastrectomie totale avec anastomose oesojéjunale sur anse en Y par voie abdominale pure, avec ou sans ouverture du hiatus reste le traitement de choix des tumeurs de type III.

## Conflits d’intérêts

Les auteurs ne déclarent aucun conflit d’intérêt.

## Contribution des auteurs

Tous les auteurs ont contribué à l’élaboration de ce travail et ont lu et approuvé la version finale du manuscrit.

## Figures and Tables

**Tableau 1:** les différents types d’interventions réalisées lors du traitement chirurgical des adénocarcinomes de la jonction  oesogastrique dans une série marocaine

Gestes réalisés	Nombre	%
Oesophagectomie transhiatale (OTH)	60	58
Gastrectomie totale (GT)	30	28.5
Oesogastrectomie polaire superieure (OGPS)	11	10.5
Lewis santy	2	2
Akiyama	1	1
Total	104	100

**Lewis-Santy**: oeso-gastrectomie +anastomose  oesogastrique intrathoracique,**Akiyama**: Oeso-gastrectomie + Anastomose oesogastrique au cou.

**Tableau 2:** Les voies d’abord utilisées lors du traitement chirurgical des adénocarcinomes de la jonction oesogastrique dans une série marocaine

Voie d’abord	Nombre	%
Abdomen seul	41	39 %
Abdomen+cou	60	58 %
Abdomen + thorax Droite	2	2 %
Abdomen + Thorax + cou	1	1 %

**Tableau 3:** les types d’anastomoses réalisées lors du traitement chirurgical des adénocarcinomes de la jonction oesogastrique dans une série marocaine

Type d’anastomose	Nombre	%
Oeso-colique, cou	44	42.5
Oeso-gastrique, cou	17	16.5
Oeso-jéjunale / anse Y	19	18
Oeso-jéjunale / anse [	2	2
Oeso-jéjunale / Henley	9	8.5
Oeso-gastrique, thorax	2	2
Oeso-gastrique, abd	11	10.5

**Tableau 4:** causes de mortalité postopératoire une série marocaine du traitement chirurgical des adénocarcinomes de la jonction oesogastrique dans

Causes	OTH	GT	OGPS	LS	Akiyama
Péritonite postopératoire Respiratoire Hémorragie Occlusion	-1 1 -	2 1 -1	-1 --	-1 --	-1 --

OTH : Oesophagectomie transhiatale , GT : Gastrectomie totale, OGPS : Oesogastrectomie polaire superieure, LS : Lewis Santy

**Tableau 5:** morbidité postopératoire lors du traitement chirurgical des adénocarcinomes de la jonction oesogastrique dans une série marocaine

Causes	OTH	LS	Akiyama	GT	OGPS
Péritonite postopératoire	2	-	-	2	-
Paralysie recurentielle	2	-	-	-	-
Fistule cervicale	3	-	-	-	-
Fistule digestive	-	-	-	2	-
Abcès sous phrénique	1	-	-	-	1
Médiastinite	-	1	-	-	-
Occlusion	-	-	-	1	-
Hémorragie	1	-	-	-	-
Pleurésie	1	-	-	1	-
Pneumopathie	2	1	1	2	2

OTH : Oesophagectomie transhiatale, GT : Gastrectomie totale, OGPS : Oesogastrectomie polaire supérieure, LS : Lewis Santy
